# Effectiveness and safety of sorafenib in the treatment of unresectable and advanced intrahepatic cholangiocarcinoma: a pilot study

**DOI:** 10.18632/oncotarget.12825

**Published:** 2016-10-23

**Authors:** Xiangji Luo, Weidong Jia, Zhiyong Huang, Xiangcheng Li, Baocai Xing, Xiaoqing Jiang, Jun Li, Anfeng Si, Tian Yang, Chunfang Gao, Wan Yee Lau, Feng Shen

**Affiliations:** ^1^ Department of Biliary Surgery, The Eastern Hepatobiliary Surgery Hospital, Second Military Medical University, Shanghai, China; ^2^ Department of Hepatobiliary Surgery, The Anhui Provincial Hospital, Hefei, China; ^3^ Department of Hepatobiliary Surgery, The Tongji Hospital, Huazhong University of Science and Technology, Wuhan, China; ^4^ Department of Hepatobiliary Surgery, The Jiangsu Provincial Peoples Hospital, Nanjing, China; ^5^ Department of Hepatobiliary Surgery, The Beijing Cancer Hospital, Beijing, China; ^6^ Department of Hepatic Surgery, The Eastern Hepatobiliary Surgery Hospital, Second Military Medical University, Shanghai, China; ^7^ Department of Laboratory Diagnosis, The Eastern Hepatobiliary Surgery Hospital, Second Military Medical University, Shanghai, China; ^8^ Faculty of Medicine, The Chinese University of Hong Kong, Shatin, Hong Kong, SAR, China

**Keywords:** intrahepatic cholangiocarcinoma, sorafenib, adverse events, disease control rate

## Abstract

Patients with unresectable and advanced intrahepatic cholangiocarcinoma (ICC) usually have short survival due to a lack of effective treatment. This multicenter, single arm, open labeled, prospective study was conducted to evaluate the effectiveness and safety of sorafenib combined with best supportive care (BSC) in these patients. We enrolled 44 patients with unresectable and advanced ICC who were treated with sorafenib (400 mg, twice daily) and BSC. The primary endpoint was disease control rate (DCR) at week 12, and the secondary endpoints included time to progression (TTP), progression-free survival (PFS), overall survival (OS), duration of therapy (DOT), and adverse events (AEs). Our results showed that the DCR was 15.9%, the median TTP was 5.6 months, and the median PFS and OS were 3.2 and 5.7 months (95% confidence interval [CI]: 2.4-4.1 months; 3.7-8.5 months), respectively. The median DOT was 1.8 months (95% CI: 1.9-3.9 months). AEs of grades 1 and 2 events occurred in 75% of patients, and AE of grade 4 (severe) was observed in 1 patient. Therefore, sorafenib in combination with BSC had an acceptable DCR and safety profile in patients with unresectable and advanced ICC.

## INTRODUCTION

Intrahepatic cholangiocarcinoma (ICC), which arises from the epithelial cells of intrahepatic bile ducts, accounts for 10%-15% of all primary hepatic malignancies [1]. Although the prevalence of ICC is still lower than hepatocellular carcinoma (HCC), the incidence and mortality associated with this malignancy are increasing worldwide [2].

Surgical resection is currently the only established treatment to achieve possible long-term survival in ICC patients [3]. Unfortunately, the resectability, curability and survival rates of ICC are extremely low because this malignancy is aggressively invasive and most patients present with unresectable and advanced diseases at their initial medical visit [4–8]. For these patients, effective treatment is very limited. Both 5-Fluorouracil (5-FU) and gemcitabine-based chemotherapy regimens have been recommended for hepatobiliary cancers in the National Comprehensive Cancer Network (NCCN) Guidelines [9], and numerous studies support the use of gemcitabine-cisplatin combination therapy for biliary tract carcinomas (BTC) as an internationally recognized standard [10–12]. Unfortunately, few studies have analyzed the impact of these treatments specifically in ICC patients. Most published reports on chemotherapy were conducted on patients with heterogeneous types of biliary tract cancers and the results were conflicting [13–15]. Furthermore, response to treatment was poor even to a combination of drugs [16]. Although there were a limited number of studies using transarterial chemoembolization (TACE) or photodynamic therapy (PDT) to treat advanced ICC, there were not enough evidences to support that these treatments to be effective [17–19]. To the best of our knowledge, there are no Food and Drug Administration (FDA)-approved targeted molecular therapies for ICC [20].

Sorafenib, a multi-kinase inhibitor of rapidly accelerated fibrosarcoma (RAF) kinase, vascular endothelial growth factor receptor -2/-3 (VEGFR-2/-3), platelet-derived growth factor receptor β (PDGFR-β), Flt3 and C-kit receptor, has been recommended in the therapy of solid cancers, such as renal cell carcinoma and HCC [21]. Unfortunately, the therapeutic effectiveness of sorafenib for ICC patients remained largely unknown. Previous studies have shown sorafenib inhibited proliferation and induced apoptosis in human ICC cell lines *in vitro*. Sorafenib also displayed antitumor activity with prolonged survival in an ICC animal model [22]. It is therefore logical to hypothesize that sorafenib is clinically effective for patients with advanced ICC.

This multicenter, single arm, open labeled, prospective study aimed to assess the effectiveness and safety of sorafenib combined with best supportive care (BSC) in patients with unresectable and advanced ICC. This is the first and only prospective pilot study to examine the use of sorafenib specifically for ICC without the confounding factor contributed by other types of cholangiocarcinoma.

## RESULTS

### Patient demographic and baseline characteristics

There were 44 patients (mean age, 56.5±10.6 y) with advanced and unresectable ICC who were enrolled in this study (Table 1). Of them, 18 patients (40.9%) had no disease-related symptoms at the inception of the study. The mean tumor diameter was 5.6±4.1 cm. Twenty patients (45.6%) had a history of surgical resection and developed recurrence which was assessed to be unsuitable for further surgical management. Eight patients (18.2%) received previous therapies for ICC including TACE (*n* = 3), chemotherapy (*n* = 3) and radiotherapy (*n* = 2), but all of them had stopped these treatments for more than 2 months prior to entry of this study (Table 1). Liver function assessments revealed that 75.0% of patients had normal alanine aminotransferase (ALT) levels, 63.4% had normal aspartate aminotransferase (AST) levels, 43.2% had normal alkaline phosphatase (ALP) levels, and 25.0% had normal gamma-glutamyl transferase (GGT) levels (Table 1).

**Table 1:** Demographics and baseline characteristics

Variable	(*n* = 44)
Age, y*	56.5±10.6
Sex^†^	
Male	25 (56.8)
Female	19 (43.2)
BMI, kg/m^2^*	23.2±3.0
SBP, mmHg*	126.0±11.8
DBP, mmHg*	81.6±9.3
ECOG†	
0	8 (18.2)
1	33 (75.0)
2	3 (6.8)
Absence of symptoms^†^	18 (40.9)
History of tumor resection^†^	20 (45.6)
Previous anti-ICC therapy^†^	
Transarterial chemoembolization	3 (6.8)
Chemotherapy	3 (6.8)
Radiotherapy	2 (4.5)
Concomitant liver diseases^†^	
Cholelithiasis	4 (9.1)
Hepatitis B virus infection	7 (15.9)
Cirrhosis	2 (4.5)
Other liver diseases	3 (6.8)
Diameters of target lesions, cm*	5.6±4.1
ALT	
Normal	33 (75.0)
>1.0-2.5ULN	7 (15.9)
>2.5-5.0ULN	1 (2.3)
>5.0-20.0ULN	0 (0.0)
>20.0ULN	0 (0.0)
Not assessed	3 (6.8)
AST	
Normal	27 (61.4)
>1.0-2.5ULN	13 (29.6)
>2.5-5.0ULN	1 (2.3)
>5.0-20.0ULN	0 (0.0)
>20.0ULN	0 (0.0)
Not assessed	3 (6.8)
ALP	
Normal	19 (43.2)
>1.0-2.5ULN	18 (40.9)
>2.5-5.0ULN	2 (4.6)
>5.0-20.0ULN	2 (4.6)
>20.0ULN	0 (0.0)
Not assessed	3 (6.8)
GGT	
Normal	11 (25.0)
>1.0-2.5ULN	19 (43.2)
>2.5-5.0ULN	3 (6.8)
>5.0-20.0ULN	7 (15.9)
>20.0ULN	1 (2.3)
Not assessed	3 (6.8)
TBIL	
Normal	31 (70.5)
Abnormal	11 (25.0)
Not assessed	2 (4.6)

* Mean ± SD

^†^ n (%)

Abbreviations: BMI, body mass index; SBP, systolic blood pressure; DBP, diastolic blood pressure; ECOG, Eastern Cooperative Oncology Group; ULN, upper limits of normal;ALT, alanine aminotransferase; AST, aspartate aminotransferase; ALP, alkaline phosphatase; GGT, gamma-glutamyl transferase; TBIL, total bilirubin.

### Response evaluation

The majority of patients (*n* = 43, 97.7%) in this study received 400 mg of oral sorafenib (Nexavar) twice daily and only one patient (2.3%) received sorafenib once daily.

During sorafenib therapy, all patients had Eastern Cooperative Oncology Group (ECOG) scores of ≤ 3.

As determined by the Response Evaluation Criteria in Solid Tumors 1.1 (RECIST 1.1) criteria, analysis of the overall imaging evaluation showed PR in one patient (4%) and SD in 15 patients (60%) at 6 weeks; CR was detected in one patient (8%) and SD in six patients (46%) at 12 weeks. At the last visit, CR was observed in one patient (2%), PR in one patient (2%), and SD in seven patients (16%). Another three patients were diagnosed with SD (15%) at the last imaging examination (Table 2). Of these patients, progressive disease (PD) was not observed within 1 year after therapy in two patients.

**Table 2:** Tumor size and response throughout the study period

	6 weeks	12 weeks
**Overall imaging evaluation ^a^**		
CR	0 (0)	1 (8)
PR	1 (4)	0 (0)
SD	15 (60)	6 (46)
PD	8 (32)	6 (46)
Unable to evaluate	1 (4)	0 (0)

^a^ n (%) for categorical data

Abbreviations: CR, complete response; PR, partial response; SD, stable disease; PD, progressive disease.

**Figure 1:**
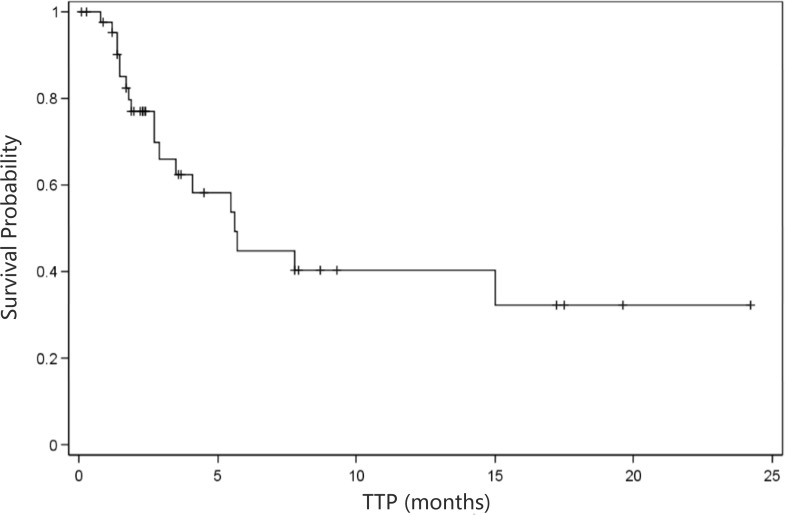
Kaplan-Meier curve of time to progression (*n* = 44) TTP, time to progression; NA, not available. Median TTP=5.6 months (95% confidence interval: 2.9 months-NA)

**Figure 2:**
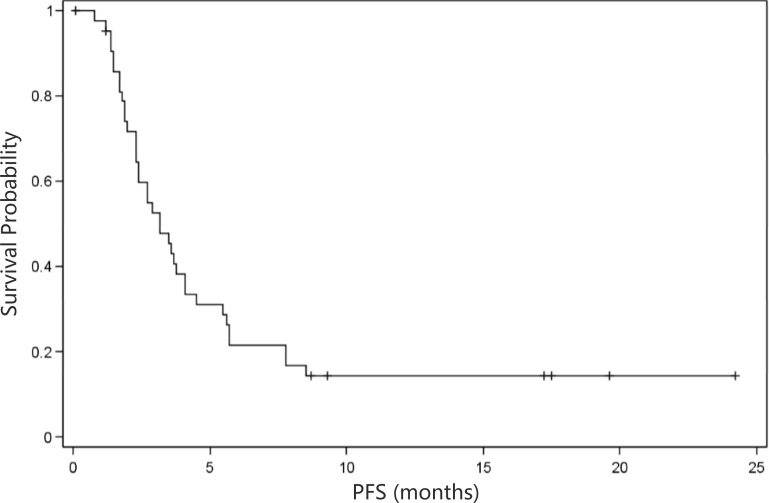
Kaplan-Meier curve of progression-free survival (*n* = 44) PFS, progression free survival. Median PFS = 3.2 months (95% confidence interval: 2.3-4.1 months)

**Figure 3:**
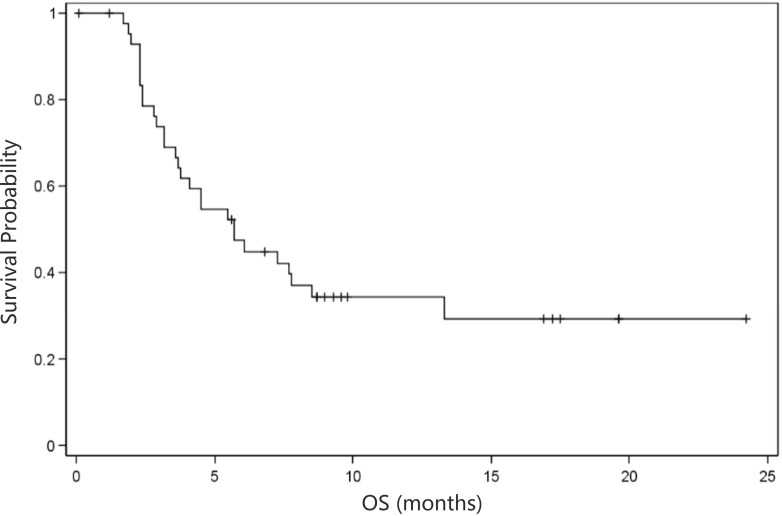
Kaplan-Meier curve of overall survival (*n* = 44) OS, overall survival. Median OS = 5.7 months (95% confidence interval: 3.7-8.5 months)

As shown in Table 3, overall evaluation showed a disease control rate (DCR) of 53.9% and a derived DCR of 15.9%. In addition, the median time to progression (TTP) was 5.6 months (95% CI: 2.9 months- NA; Figure 1), and the median progression-free survival (PFS) was 3.2 months (95% CI: 2.3- 4.1 months; Figure 2). In addition, the median overall survival (OS) was 5.7 months (95% CI: 3.7- 8.5 months; Figure 3).

**Table 3:** Summary of the disease control rate (DCR) at 12 weeks after treatment initiation

Parameters	N (%)	95% CI
DCR		
Overall efficacy (N = 13)	7 (53.9)	25.1 -80.8
DCR (derived)*		
Overall efficacy (N = 44)	7 (15.91)	6.64-30.07

* Rules for identifying the derived DCR.

1. If PD was not achieved within 12 weeks and there was no evaluation at week 12, the latest results of evaluation after 12 weeks were moved forward to week 12;

2. If patients died within 12 weeks and there was no evaluation at week 12, the outcome at week 12 was defined as PD;

3. If PD was achieved within 12 weeks and there was no evaluation at week 12, the outcome at week 12 was defined as PD; and

4. If PD was noted at the second evaluation (week 6) and there was no evaluation thereafter, the outcome at week 12 was defined as PD.

### Safety outcomes

The duration of therapy (DOT) was 2.9±3.4 months with a median of 1.8 months (95% CI: 1.9-3.9 months; data not shown). Based on the National Cancer Institute’s Common Terminology Criteria for Adverse Events (AEs) version 4.0 (NCI-CTCAE 4.0), AEs were observed in 33 patients (75%) treated with sorafenib. The most commonly observed AEs were classified as grades 1 or 2 which and included diarrhea, hand and foot skin reaction, and fatigue. Grade 4 severe AE (SAE) (hand and foot skin reaction) was found only in one patient. Of grade 3 AEs, diarrhea accounted for 13.6%, hand and foot skin reaction 2.3%, fatigue 2.3%, rash 4.5%, loss of appetite 2.3%, hair loss 2.3%, increase in transaminase 2.3%, stomatitis 2.3%, leucopenia 2.3%, low back radiating pain 2.3%, waist and abdominal pain 2.3%, and scrotal skin lesions 2.3% (Table 4).

**Table 4:** Summary of adverse events by CTCAE grade

		CTCAE grade			
Adverse events	Total (N = 44)	Grade I	Grade II	Grade III	Grade IV
Diarrhea	19 (43.2%)	4 (9.1%)	11 (25.0%)	6 (13.6%)	0 (0%)
Hand and foot skin reaction	15 (34.1%)	4 (9.1%)	14 (31.8%)	1 (2.3%)	1 (2.3%)
Fatigue	15 (34.1%)	9 (20.5%)	9 (20.5%)	1 (2.3%)	0 (0%)
Rash	11 (25.0%)	6 (13.6%)	4 (9.1%)	2 (4.5%)	0 (0%)
Loss of appetite	11 (25.0%)	5 (11.4%)	6 (13.6%)	1 (2.3%)	0 (0%)
Thrombocytopenia	6 (13.6%)	3 (6.8%)	4 (9.1%)	0 (0%)	0 (0%)
Hair loss	5 (11.4%)	3 (6.8%)	2 (4.5%)	1 (2.3%)	0 (0%)
Nausea	4 (9.1%)	2 (4.5%)	2 (4.5%)	0 (0%)	0 (0%)
Elevated transaminase	4 (9.1%)	3 (6.8%)	0 (0%)	1 (2.3%)	0 (0%)
Stomatitis	3 (6.8%)	1 (2.3%)	2 (4.5%)	1 (2.3%)	0 (0%)
Leukopenia	3 (6.8%)	1 (2.3%)	1 (2.3%)	1 (2.3%)	0 (0%)
Constipation	2 (4.5%)	2 (4.5%)	0 (0%)	0 (0%)	0 (0%)
Fever	2 (4.5%)	1 (2.3%)	1 (2.3%)	0 (0%)	0 (0%)
Hypertension	2 (4.5%)	1 (2.3%)	1 (2.3%)	0 (0%)	0 (0%)
Joint pain	2 (4.5%)	1 (2.3%)	1 (2.3%)	0 (0%)	0 (0%)
Oral ulcer	2 (4.5%)	1 (2.3%)	1 (2.3%)	0 (0%)	0 (0%)
Vomiting	2 (4.5%)	1 (2.3%)	1 (2.3%)	0 (0%)	0 (0%)
Elevated bilirubin	1 (2.3%)	0 (0%)	1 (2.3%)	0 (0%)	0 (0%)
Low blood chloride	1 (2.3%)	1 (2.3%)	0 (0%)	0 (0%)	0 (0%)
Hyponatremia	1 (2.3%)	1 (2.3%)	0 (0%)	0 (0%)	0 (0%)
Lipsotrichia	1 (2.3%)	1 (2.3%)	0 (0%)	0 (0%)	0 (0%)
Ear discomfort	1 (2.3%)	0 (0%)	1 (2.3%)	0 (0%)	0 (0%)
Jaundice, elevated bilirubin	1 (2.3%)	0 (0%)	1 (2.3%)	0 (0%)	0 (0%)
Lymphopenia	1 (2.3%)	1 (2.3%)	0 (0%)	0 (0%)	0 (0%)
Haematemesis	1 (2.3%)	0 (0%)	1 (2.3%)	0 (0%)	0 (0%)
Yellow skin / sclera	1 (2.3%)	0 (0%)	1 (2.3%)	0 (0%)	0 (0%)
Anemia	1 (2.3%)	1 (2.3%)	0 (0%)	0 (0%)	0 (0%)
Body weight loss	1 (2.3%)	1 (2.3%)	0 (0%)	0 (0%)	0 (0%)
Headache	1 (2.3%)	0 (0%)	1 (2.3%)	0 (0%)	0 (0%)
Bloody stools	1 (2.3%)	0 (0%)	1 (2.3%)	0 (0%)	0 (0%)
Lower back radiating pain	1 (2.3%)	0 (0%)	0 (0%)	1 (2.3%)	0 (0%)
Waist and abdominal pain	1 (2.3%)	0 (0%)	0 (0%)	1 (2.3%)	0 (0%)
Scrotal skin lesions	1 (2.3%)	0 (0%)	0 (0%)	1 (2.3%)	0 (0%)
Elevated lipase	1 (2.3%)	1 (2.3%)	0 (0%)	0 (0%)	0 (0%)
Elevated direct bilirubin	1 (2.3%)	1 (2.3%)	0 (0%)	0 (0%)	0 (0%)
Elevated total protein	1 (2.3%)	1 (2.3%)	0 (0%)	0 (0%)	0 (0%)

For grades 0-2 AEs, no dose modification was required. Patients with grade 3 AEs had their doses modified to sorafenib 400 mg sorafenib once daily. Patients with grade 4 hematologic AEs discontinued therapy until the AE resolved to grade 2 or lower. In total, 11 patients had dose modifications, including seven patients that had their doses decreased as well as four patients that were readministered sorafenib following discontinuation. Eleven patients with nonhematologic grade 4 AEs completely discontinued the therapy.

## DISCUSSION

A previous study showed sorafenib had *in vivo* antitumor activity and it prolonged survival of mice harboring peritoneally disseminated ICC [22]. However, clinical evidence to show the effectiveness of oral sorafenib in patients with unresectable and advanced ICC is still lacking. In this prospective open-labeled study, the effectiveness and safety were studied in 44 ICC patients who received sorafenib combined with BSC. The overall DCR was 53.9%, the median TTP was 5.6 months, the median PFS was 3.2 months, the median OS was 5.7 months, and the DOT was 2.9±3.4 months. In addition, most AEs experienced by the patients were grades 1 or 2 which included diarrhea, hand and foot skin reaction, and fatigue. Thus, sorafenib combined with BSC had acceptable disease control and safety profile for ICC patients.

Two randomized studies and one meta-analysis have shown the effectiveness of doublet gemcitabine-cisplatin in patients with heterogeneous types of biliary tract cancers at advanced stage. Although exploratory sub-group analysis by Valle et al. [11, 12] showed benefits of the gemcitabine-cisplatin combination therapy in all patients despite primary tumor sites, and no significant differences in treatment effect were observed in OS and PFS. Knowledge on current treatment for unresectable and advanced ICC is still limited and few studies have specifically focused on ICC. Indeed, only 28 patients of 83 (33.7%) had ICC in the study by Okusaka et al. [10] and only 21.9% in the study by Valle et al. [12]. For unresectable ICC, the NCCN Guidelines recommend gemcitabine/cisplatin combination therapy, fluoropyrimidine-based or gemcitabine-based chemotherapy regimens, fluoropyrimidine chemoradiation or supportive care [9]. However, clinical studies comparing various chemotherapeutic agents and their combinations showed conflicting results in patients with unresectable ICC. In a study using hepatic arterial infusion comparing floxuridine alone or with bevacizumab, the median survivals were 39.3 and 28.5 months, respectively [23]. In another study, the DCR in patients with advanced and unresectable ICC treated with capecidtabine plus cisplatin was 41.5% [24]. Other studies which included patients with ICC and other types of cholangiocarcinoma reported gemcitabine induced response rates of 0 to 36% and median survivals of 4.6 to 14.0 months were reported, while a study using mitomycin C, cisplatin, taxane and irinotecan (CPT-11) reported a response rate of 10% and median survival rates of 4.5 to 6.1 months [25–28].

Supplemental Table 1 describes and compares the effects of various treatments for ICC. In patients with locally advanced or metastatic BTC, those treated with gemcitabine-cisplatin combination therapy, the median OS were 11.7 [11] and 11.2 [10] months, the median PFS were 8.0 [11] and 5.8 [10] months and the DCR were 81.4% [11] and 68.3% [10]. Although treatment with sorafenib resulted in lower median PFS and OS of 3.2 and 5.7 months, respectively, and a DCR of 53.9%, direct comparisons between the results of these studies to suggest sorafenib to be inferior to chemotherapy is not appropriate because of differences in patient selection and case-mix. Furthermore, these studies were conducted in patients with heterogeneous types of biliary tract cancers which included hilar cholangiocarcinoma, extrahepatic cholangiocarcinoma, gallbladder cancer, Vater’s ampulla cancer, and ICC. There were no attempts for these studies to separately report on the effectiveness of systemic chemotherapy on the different types of biliary tract cancers [10, 11]. This point is particularly important given that ICC exhibits different mechanisms of carcinogenesis mechanisms, molecular profiles, and biologic behaviors from the other biliary tract cancers, such as hilar cholangiocarcinoma, extrahepatic cholangiocarcinoma, and gallbladder cancer [29, 30]. A recent study revealed that *KRAS* and *TP53* mutations were relatively common in cholangiocarcinoma, particularly in extrahepatic cholangiocarcinoma, while *IDH1/2* and *BRAF* mutations were considerably more prevalent in ICC [31]. Notably, the authors also suggested that the emerging data pointed to an overlapping molecular profile between subclasses of ICC and HCC (hepatocellular carcinoma). The difference in genetic mutations related to different signal pathways between ICC and other biliary tract cancers might contribute to a varied response to chemotherapy and molecular targeted therapy. Although there are no sufficient data to indicate a difference in response to chemotherapy between ICC and other biliary tract cancers, some studies have suggested such a difference exists [32]. There is a study which showed the overall response rate to be better in patients with gallbladder cancer than for those with ICC (54.4% *versus* 21.4%, respectively) [33].

ICC is generally considered as a cancer of high malignancy with less than 4 months survival in patients not treated with surgery, chemotherapy, or radiotherapy [34, 35], As our study showed a median OS in patients treated with sorafenib to be 5.7 months, sorafenib should be considered as a treatment options for advanced ICC. The overlapping molecular profile between subclasses of ICC and HCC as mentioned previously suggests a possible role of sorafenib in selected ICC patients [31].

Apart from a few case reports which studied the effect of sorafenib in patients with ICC, this is the first prospective pilot study to examine the use role of sorafenib in ICC patients. In a case report of two patients with advanced ICC who were treated with sorafenib, LaRocca et al showed disease control for >4 months. One patient received oxaliplatin combined with gemcitabine after sorafenib therapy survived for 16 weeks and another patient achieved stable survival for 24 weeks before the case report [36]. In another case report, sorafenib extended the survival of a patient with advanced ICC for more than 4 years [37]. The 12-week DCR of 53.9% and a median OS of 5.7 months in the present study are better than those reported in phase II clinical trials on sorafenib in patients with all types of cholangiocarcinoma [13, 14, 36–38]. These studies also suggested that addition of sorafenib did not confer any survival benefits but was associated with increased toxicity [14, 15]. Differences in patient populations and types of cholangiocarcinoma (extrahepatic cholangiocarcinoma [ECC] *vs*. ICC) can account for the the difference in response rates to sorafenib, as cholangiocarcinomas developed from different origins show different biological characteristics, chemosensitivies, and prognoses [39, 40]. Thus, further studies comparing the effectiveness of sorafenib plus BSC with BSC alone in patients with advanced ICC are required.

As reported in the previous phase II studies of sorafenib in cholangiocarcinoma, most of AEs in our present study were grades 1 and 2, which consisted mainly of diarrhea, hand and foot skin reaction and fatigue (Supplemental Table 2) [13,38]. Of note, only one patient experienced a grade 4 SAE with severe hand and foot skin reaction. Thus, the patients enrolled in this study showed favorable tolerance to sorafenib.

This study had limitations. First, it is a non-controlled, single arm study. The prognostic data of other treatments used for comparison were based on previously published reports. Second, patients who were included in this study to receive sorafenib were based on their own free will after discussion with their clinicians. Third, the patients were highly selected given the extensive inclusion and exclusion criteria. Finally, the number of non-evaluated patients at 6-weeks (19/44) and at 12-weeks (32/44) were high.

In conclusion, this prospective pilot study showed sorafenib combined with BSC had a modest effect and was safe for patients with advanced ICC. Further properly conducted studies are required to define the role of sorafenib in patients with unresectable ICC.

## MATERIALS AND METHODS

### Study design and patients

This is a multicenter, single arm, open labeled, prospective study, conducted in the Eastern Hepatobiliary Surgery Hospital of Second Military Medical University, the Anhui Provincial Hospital, the Tongji Hospital of Huazhong University of Science and Technology, the Jiangsu Provincial Peoples’ Hospital, and the Beijing Cancer Hospital. ICC was diagnosed by histopathological or cytological examination.

The inclusion criteria were: (1) age ≥18 years; (2) expected survival of ≥12 weeks; (3) advanced ICC assessed as inoperable by three experienced hepatobiliary surgeons in each center; (4) an ECOG performance status score of 0-2; (5) normal bone marrow, liver, and kidney function before the study; (6) negative serum/urine pregnancy test within 7 days before the study for women of child-bearing age; and (7) ability to take oral medication. In addition, only patients with RECIST-evaluable disease at baseline were included.

The exclusion criteria were: (1) other malignancies; (2) associated major organ dysfunction or failure; (3) received systemic chemotherapy, molecular targeted therapy (including sorafenib) or biological response modifiers within 2 months of the study; (4) human immunodeficiency virus (HIV) infection or other severe active infection (> grade 2 according to the NCI-CTCAE 4.0); (5) severe non-healing wound, gastrointestinal bleeding, fracture, major surgery, open biopsy and severe trauma within 4 weeks of the study; (6) thrombosis or embolic events within 6 months of the study; (7) uncontrolled ascites; (8) suspected or known allergy to drugs used in the study; (9) bone marrow, stem cell or other organ transplantation; and (10) the presence of mixed hepato-cholangiocarcinoma.

The assumed DCR was 35% and invalid DCR was 15%. Using a ‘Hern single stage design, given α=0.05 and a power of 90%, a sample of at least 38 patients were needed. If 10 patients were observed in the study to reach the disease control, the DCR would be regarded as >15%; considering of a 15% drop-off, the total number needed for enrolled was 45.

During the study period, 44 consecutive patients who met the above inclusion and exclusion criteria were recruited into the study. This study was approved by the Institutional Ethics Committees of the respectively centers. Informed consent was obtained from all the patients prior to enrollment.

### Treatment with sorafenib and BSC

Patients were treated with 400 mg of oral sorafenib, twice daily. Treatment was continued until disease progression, intolerable AEs, or patient withdrawal from the study. In the event of any intolerable toxicity as graded according to the NCI-CTCAE 4.0, the dose of sorafenib was reduced to 400 mg daily or 400 mg every other day or the therapy was discontinued. The dose of sorafenib was readjusted to 400 mg twice daily once the adverse events resolved. All patients who withdrew from this study due to toxicity were followed-up until the toxicity resolved and, thereafter, until they died.

The BSC used in the study included protection of liver function, relief of symptoms, and nutritional support.

### Patient evaluation and follow-up

Patients were followed-up once every 3 weeks (±3 days) within the first 1-3 months, and once every 6 weeks (±7 days) thereafter. Imaging examinations (chest/abdominal computed tomography (CT) scans or magnetic resonance imaging (MRI); cranial imaging or bone scans, if necessary) were performed within 4 weeks before initiation of sorafenib therapy (the screening period) and once every 6 weeks during sorafenib therapy. Three experienced radiologists independently evaluated the imaging data, and any controversies on imaging findings were resolved by discussion. The therapeutic effectiveness was re-evaluated within 30 days after the last therapy. AEs and any alterations in dosage of sorafenib were recorded. During follow-up after therapy, all severe AEs (SAEs) were recorded. All patients who did not attend follow-up were contacted by phone by a research nurse.

### Primary and secondary endpoints

The primary endpoint was DCR at week 12. Disease control was defined as the proportion of patients who had no disease progression (i.e., those with a CR, PR, or SD as determined by the RECIST 1.1 criteria), and who still received sorafenib therapy. Thus, DCR was calculated as the rate of patients with CR/PR/SD who still received sorafenib over the whole evaluated patient population. The derived DCR was determined as follows: (1) if PD was not achieved within 12 weeks and there was no evaluation at week 12, the latest results of evaluation after 12 weeks were moved forward to week 12; (2) if patients died within 12 weeks and there was no evaluation at week 12, the outcome at week 12 was defined as PD; (3) if PD was achieved within 12 weeks and there was no evaluation at week 12, the outcome at week 12 was defined as PD; and (4) if PD was noted at the second evaluation (week 6) and there was no evaluation thereafter, the outcome at week 12 was defined as PD.

In addition, the following secondary endpoints were determined: TTP which was defined as the time interval from initiation of sorafenib to disease progression as determined by imaging examinations; PFS which was defined as the time interval from initiation of sorafenib to disease progression as determined by imaging examinations or death; OS which was defined as the time interval from initiation of sorafenib to death due to any cause; DOT; and safety.

### Statistical analysis

Patients’ demographic, clinical data, therapy/intervention and outcomes were summarized as mean± standard deviation (SD) for continuous data with normal distributions, median (IQR: 1^st^ and 3^rd^ quartiles) for those without normal distribution, and n (%) for categorical data. The OS, TTP and PFS were analyzed and represented using the Kaplan-Meier curves. All analyses were performed using the SPSS Medical Pack for Windows (version 11.0; SPSS, Chicago, IL, USA). A *p* < 0.05 was considered as statistically significant.

## SUPPLEMENTARY MATERIAL TABLES



## Abbreviations

5-FU: 5-Fluorouracil
AEs: adverse events
BSC: best supportive care
CR: complete remission
CT: computed tomography
DOT: duration of therapy
DCR: disease control rate
ECOG: Eastern Cooperative Oncology Group
ECC: extrahepatic cholangiocarcinoma
FDA: Food and Drug Administration
HCC: hepatocellular carcinoma
HIV: human immunodeficiency virus
ICC: Intrahepatic cholangiocarcinoma
MRI: Magnetic Resonance Imaging
NCCN: National Comprehensive Cancer Network
OS: overall survival
PR: partial remission
PDT: photodynamic therapy
PDGFR-β: platelet-derived growth factor receptor β
PFS: progression-free survival
RECIST: Response Evaluation Criteria in Solid Tumors
RAF: rapidly accelerated fibrosarcoma
SAEs: severe AEs
SD: stable disease
SD: standard deviation
TTP: time to progression
TACE: transarterial chemoembolization
VEGFR-2/-3: vascular endothelial growth factor receptor-2/-3
