# Supplementary material for: Effectiveness and safety of sorafenib in the treatment of unresectable and advanced intrahepatic cholangiocarcinoma: a pilot study
Source: Oncotarget. 2016 Oct 23;8(10):17246–57. doi: 10.18632/oncotarget.12825 (PMC5370037; doi:10.18632/oncotarget.12825)
Supplement: Supplementary file 1 [file oncotarget-08-17246-s001.pdf]

Effectiveness and safety of sorafenib in the treatment of unresectable and advanced intrahepatic cholangiocarcinoma: a pilot study

Supplementary Material

Supplemental Table 1. Summary of the median time for OS, TTP, and PFS among the studies assessing various treatments in patients with ICC

			OS (months)		TTP (months)	PFS (months)	
**Authors**	**n**	**Treatment**	**Median (95% CI)**	**HR (95% CI)**	**Median (95% CI)**	**Median (95% CI)**	**HR (95% CI)**
This study	44	400 mg of oral Sorafenib, twice daily	5.7 (3.7-8.5)		5.6 (2.9-NA)	3.2 (2.4-4.1)	
Moehler M, 2014	97	49 Gemcitabine+Sorafenib	8.4			3.0 (1.8-7.2)	1.28 (0.91-2.02)
		48 Gemcitabine + Placebo	11.2, p=0.775			4.9 (3.5-7.7, p=0.859)	
Valle, 2014	493	Cisplatin +Gemcitabine<br>Gemcitabine	No data for overall (a meta study)	0.65 (0.54-0.78)			0.64 (0.53-0.76)
Lee JK, 2013	39	Gemcitabine+ cisplatin+ sorafenib	14.4 (11.6-19.2)		8.2 (6.5-12)	6.5 (3.5 - 8.3)	
Kim YI, 2013	92	67 Capecitabin+Cisplatin vs. 25 Capecitabin+Cisplatin+CCRT	6.2 vs. 9.3, p=0.048			1.9 vs. 4.3, p=0.001	
		Overall	7.9 (5.8 - 10)			2.6 (2.0-3.2)	

El-Khoueiry AB, 2011	31	Sorafenib	9 (4-12)			3 (2-4)	
Bengala C, 2010	46	Sorafenib	4.4 (range: 0-22)			2.3 (range: 0-12)	
Okusaka, 2010	83	41Cisplatin + Gemcitabine<br>42 Gemcitabine	11.2<br>7.7	0.69 (0.41-1.13)		5.8<br>3.7	0.66 (0.41-1.05)
Valle, 2010	410	204 Cisplatin + Gemcitabine vs. 206 Gemcitabine	11.7 (9.5-14.3) vs. 8.1 (7.1-8.7)	0.64 (0.52-0.80)		8.0 (6.6-8.6) vs. 5.0 (4.0-5.9)	0.63 (0.51 - 0.77)
Okusaka, 2006	40	Gemcitabine	7.6 (5.4-9.3)			2.6 (1.7 - 3.8)	
Tsavaris, 2004	30	Gemcitabine	14 (13.3-16)		7 (4.5-6.0)	7 (range: 5-34)	
Alberts, 2002	36	Irinotecan	1.6 (range: 1.3-11.6)				
	34	Irinotecan	11 biliary patients: 6.1 (range: 5.9-8.7)<br>23 gallbladder patients: 7.0 (range: 3.7-8.4)			11 biliary patients: 3.2 (1.4-4.4)<br>23 gallbladder patients: 2.7 (1.7-3.3)	
Penz, 2001	32	Gemcitabine	11.5 (range: 3-24)			5.6 (range: 1.8-13)	

Abbreviations: OS, overall survival; TTP, time to progression; PFS, progression-free survival; HR, hazard ration, CI, confidence intervals; CCRT, computerized controlled radiation therapy.

Supplemental Table 2. Summary of the adverse events among three studies assessing various treatments in patients with ICC

Items of adverse events	This study	Bengala C, 2010	Anthony B, 2011
	**N=44**	**N=46**	**N=31**
**Rash**	11 (25.0)	16 (35)	2 (6)
**Hand and foot skin reaction**	15 (34.1)	8 (17)	4 (13)
**Gastrointestinal**			
Diarrhea	19 (43.2)	4 (8)	-
Stomatitis	3 (6.8)	4 (8)	-
Nausea/vomiting	6 (13.6)	4 (8)	4 (13)
Liver enzyme	-	3(6)	-
**Hematological**			
Anemia	1 (2.3)	3 (6)	-
Thrombocytopenia	6 (13.6)	11 (24)	-
Lymphopenia	1 (2.3)	-	-
Haematemesis	1 (2.3)	-	-
Leukopenia	3 (6.8)	-	-
**Systemic symptoms**			
Fatigue	15 (34.1)	15 (33)	2 (6)
**Infection**	-	1 (2)	-
**Cardiovascular**			
Deep vein thrombosis	-	1 (2)	-
Pulmonary embolism	-	2 (4)	-
Cardiac ischemia	-	1 (2)	-
**Coagulation (DIC)**	-	1 (2)	-
**Hemorrhage/bleeding**	-	1 (2)	-
**Gastrointestinal bleeding**	-	-	2 (6)
**Metabolic/laboratory**	−	6 (13)	−
**Non-hematologic**			
Venous thromboembolism	-	-	3 (10)
AST/ALT elevation	-	-	3 (10)
Elevated lipase	1 (2.3)	-	-
Elevated bilirubin	1 (2.3)	-	4 (13)
Elevated direct bilirubin	1 (2.3)	-	-
Elevated total protein	1 (2.3)	-	-
Elevated transaminase	4 (9.1)	-	-
Loss of appetite	11 (25.0)	-	-
Hair loss	5 (11.4)	-	-
Constipation	2 (4.5)	-	-
Fever	2 (4.5)	-	-
Hypertension	2 (4.5)	-	2 (6)

Joint pain	2 (4.5)	-	-
oral ulcer	2 (4.5)	-	-
Low blood chloride	1 (2.3)	-	-
Hypernatremia	1 (2.3)	-	-
Leptotrichia	1 (2.3)	-	-
Ear discomfort	1 (2.3)	-	-
Jaundice, elevated bilirubin	1 (2.3)	-	-
Yellow skin / sclera	1 (2.3)	-	-
Body weight loss	1 (2.3)	-	-
Headache	1 (2.3)	-	-
Bloody stools	1 (2.3)	-	-
Lower back radiating pain	1 (2.3)	-	-
Waist and abdominal pain	1 (2.3)	-	3 (10)
Scrotal skin lesions	1 (2.3)	-	-

Results were summarized as n(%).
